# Can Renal and Bladder Ultrasound Replace Computerized Tomography Urogram in Patients Investigated for Microscopic Hematuria?

**DOI:** 10.1016/j.juro.2018.04.065

**Published:** 2018-11

**Authors:** Wei Shen Tan, Rachael Sarpong, Pramit Khetrapal, Simon Rodney, Hugh Mostafid, Joanne Cresswell, James Hicks, Abhay Rane, Alastair Henderson, Dawn Watson, Jacob Cherian, Norman Williams, Chris Brew-Graves, Andrew Feber, John D. Kelly, DETECT I Trial Collaborators

**Affiliations:** Division of Surgery and Interventional Science (SR), University College London, London, United Kingdom; Surgical and Interventional Trials Unit (RS, NW, CB-G), University College London, London, United Kingdom; Department of Urology, University College London Hospital (WST, PK, JDK), London, United Kingdom; University College London Cancer Institute (SR, AF), London, United Kingdom; Department of Urology, Royal Surrey County Hospital (HM), Guildford, United Kingdom; Department of Urology, James Cook University Hospital (JC), Middlesbrough, United Kingdom; Department of Urology, Western Sussex Hospitals NHS Foundation Trust, Worthing Hospital (JH), Worthing, United Kingdom; Department of Urology, East Surrey Hospital (AR), Redhill, United Kingdom; Department of Urology, Maidstone Hospital (AH), Maidstone, United Kingdom; Department of Urology, Pennine Acute Hospitals NHS Trust, North Manchester General Hospital (JC), Crumpsall, United Kingdom

**Keywords:** bladder neoplasms, kidney neoplasms, hematuria, tomography, x-ray computed, cystoscopy, AUA, American Urological Association, CT, computerized tomography, CTU, CT urogram, DETECT, Detecting Bladder Cancer Using the UroMark Test, NHS, National Health Service, NPV, negative predictive value, PPV, positive predictive value, RBUS, renal and bladder ultrasound, UTUC, upper tract urothelial carcinoma

## Abstract

**Purpose:**

Computerized tomography urogram is recommended when investigating patients with hematuria. We determined the incidence of urinary tract cancer and compared the diagnostic accuracy of computerized tomography urogram to that of renal and bladder ultrasound for identifying urinary tract cancer.

**Materials and Methods:**

The DETECT (Detecting Bladder Cancer Using the UroMark Test) I study is a prospective observational study recruiting patients 18 years old or older following presentation with macroscopic or microscopic hematuria at a total of 40 hospitals. All patients underwent cystoscopy and upper tract imaging comprising computerized tomography urogram and/or renal and bladder ultrasound.

**Results:**

A total of 3,556 patients with a median age of 68 years were recruited in this study, of whom 2,166 underwent renal and bladder ultrasound, and 1,692 underwent computerized tomography urogram in addition to cystoscopy. The incidence of bladder, renal and upper tract urothelial cancer was 11.0%, 1.4% and 0.8%, respectively, in macroscopic hematuria cases. Patients with microscopic hematuria had a 2.7%, 0.4% and 0% incidence of bladder, renal and upper tract urothelial cancer, respectively. The sensitivity and negative predictive value of renal and bladder ultrasound to detect renal cancer were 85.7% and 99.9% but they were 14.3% and 99.7%, respectively, to detect upper tract urothelial cancer. Renal and bladder ultrasound was poor at identifying renal calculi. Renal and bladder ultrasound sensitivity was lower than that of computerized tomography urogram to detect bladder cancer (each less than 85%). Cystoscopy had 98.3% specificity and 83.9% positive predictive value.

**Conclusions:**

Computerized tomography urogram can be safely replaced by renal and bladder ultrasound in patients who have microscopic hematuria. The incidence of upper tract urothelial cancer is 0.8% in patients with macroscopic hematuria and computerized tomography urogram is recommended. Patients with suspected renal calculi require noncontrast renal tract computerized tomography. Imaging cannot replace cystoscopy to diagnose bladder cancer.

Hematuria is a cardinal clinical symptom with an associated risk of urinary tract cancer. The risk of malignancy in patients who present with macroscopic hematuria is 20.4% and in comparison the risk of malignancy is 5.2% in patients who present with microscopic hematuria.^1^ Bladder cancer is the most common cancer detected in microscopic hematuria cases, accounting for 4.8% of those investigated, while renal cancer and UTUC are less common with an incidence of 0.3% and 0.1%, respectively.^1^

Recommendations on who should be investigated for microscopic hematuria differ across guideline bodies.^2^ While there is a resounding consensus that cystoscopy remains the investigation of choice to visualize the bladder, there is a lack of consensus on the optimal upper tract imaging. RBUS and CTU are the most commonly used imaging modalities. The AUA recommends performing CTU for macroscopic and microscopic hematuria while the United Kingdom NICE (National Institute for Health and Care Excellence) and the ACP (American College of Physicians®) do not specify a recommended imaging modality.^3^, ^4^, ^5^ Similarly the role of upper tract imaging in patients newly diagnosed with bladder cancer also differs among guidelines.^6^

CTU has the highest diagnostic performance for identifying upper tract disease. A meta-analysis suggested that CTU achieves 93% sensitivity and 99% specificity for UTUC.^7^ However, the diagnostic performance of CTU should be balanced against the risk due to intravenous contrast medium. Intravenous contrast administration is associated with a 3% risk of contrast induced nephropathy in patients at high risk, defined as an estimated glomerular filtration rate of 30 to 59 ml/minute/1.73 m^2^. Prophylaxis hydration was shown to be ineffective.^8^, ^9^ In addition, exposure to ionizing radiation is carcinogenic and there is a risk of anaphylactic reaction, although it is rare.^10^, ^11^

The DETECT I study (ClinicalTrials.gov NCT02676180) is a prospective, multicenter, observational study prospectively recruiting patients referred from primary care physicians to urology departments for investigation following the presentation of hematuria.^12^ We report the incidence of upper tract disease and bladder cancer in patients with macroscopic and microscopic hematuria as well as the diagnostic ability of CTU and RBUS to identify upper tract cancer to determine whether CTU can be safely replaced by RBUS in patients who present with microscopic hematuria.

## Patients and Methods

Between March 2016 and June 2017 DETECT I recruited patients from 40 hospitals throughout the United Kingdom with 1-stop hematuria investigation clinics. All patients were referred to secondary care following presentation with hematuria. Macroscopic hematuria was defined as visible hematuria reported by the patient or the primary care physician. Microscopic hematuria was defined as 1 or greater red blood cells on urine dipstick on 2 or more occasions.^13^ The study inclusion criterion was male or female patients 18 years old or older who were willing to provide consent. All patients underwent cystoscopy and upper tract imaging within 12 weeks after study registration. Determining the diagnostic accuracy of RBUS and CTU represents a post hoc analysis.

The study protocol was approved by Health Research Authority, North West Liverpool Central Research Ethics Committee, on March 2016 (Integrated Research Application System Project ID 179245, Research Ethics Committee reference 16/NW/0150). The full study protocol has been previously described.^12^

Medical history was obtained and physical examination was performed in all patients. Patient demographics were collected, including age, gender, occupation, ethnicity and smoking history. Patients with a suspicion of bladder cancer underwent transurethral resection of bladder cancer or bladder biopsy under general anesthesia. The reference standard for bladder cancer was histopathological examination as classified according to the WHO TNM tumor classification.^14^ Bladder cancer was risk stratified based on clinicopathological features in the EAU (European Association of Urology) risk classification.^15^ Upper tract imaging comprised 1 or more radiological imaging modalities, including CTU and/or RBUS.

DETECT I is a pragmatic, observational design study. The choice of upper tract imaging and the decision to perform more than 1 imaging modality was determined according to local hospital guidelines. Renal cancer and UTUC were confirmed by histopathological examination when nephrectomy or renal biopsy was performed except in a small number of renal cancers, which were treated with active surveillance without biopsy. Renal calculi diagnosed on CTU served as the reference standard.

Continuous data, including the mean, median, IQR and 95% CI, are reported using descriptive statistics. Categorical variables were compared by the chi-square test. The t-test was applied to compare continuous variables. A normal distribution was assumed. The sensitivity, specificity, PPV and NPV of correctly identifying bladder or upper tract cancer were calculated. IBM® SPSS®, version 22 was used to perform all statistical analysis. Statistical significance was considered at p <0.05.

This report adhered to the STROBE (Strengthening the Reporting of Observational studies in Epidemiology) guidelines. This study was registered with ClinicalTrials.gov (number NCT02676180).

## Results

### Patient Demographics

The figure shows a flow diagram of the 3,556 patients with a median age of 68 years (IQR 57–76) who were recruited in the study. The supplementary table (http://jurology.com/) lists patient demographics. The overall incidence of urinary tract cancer was 10.0%, including 8.1% for bladder cancer, 1.0% for renal cancer and 0.5% for UTUC. RBUS was performed in 2,166 patients (60.9%) and CTU was done in 1,692 (47.6%). In 475 patients (13.2%) RBUS and CTU were performed.

**Figure 1:**
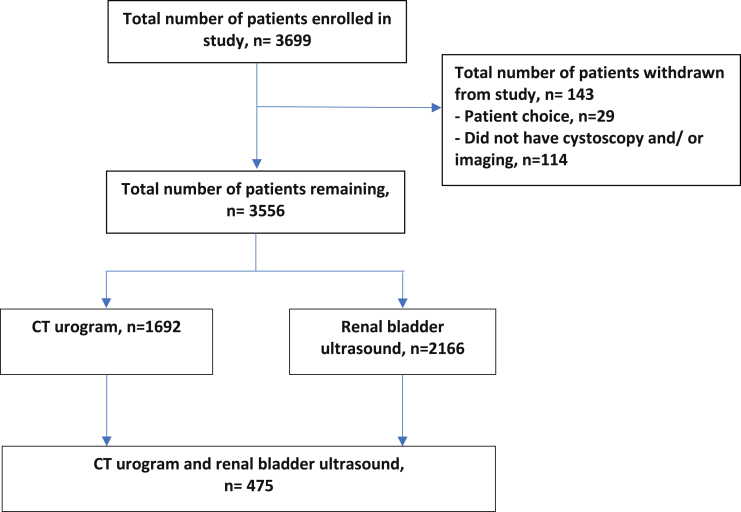
Flow diagram of patients recruited in study

### Urinary Tract Disease Incidence

The supplementary table (http://jurology.com/) shows the incidence of urinary tract cancer and renal stones stratified by the presentation of microscopic and macroscopic hematuria. Overall 33 patients (2.7%) investigated for microscopic hematuria had a diagnosis of bladder cancer, 5 (0.4%) had renal cancer and 55 (4.4%) had renal calculi. No patient with nonvisible hematuria was diagnosed with UTUC.

By comparison patients with macroscopic hematuria had a higher incidence of urinary tract disease than those with microscopic hematuria. Of the patients investigated for macroscopic hematuria 255 (11.0%) had bladder cancer, 32 (1.4%) had renal cancer and 18 (0.8%) had a diagnosis of UTUC. A diagnosis of renal calculi was confirmed in 215 patients (9.3%).

### Renal and Bladder Ultrasound, and Computerized Tomography Urogram Diagnostic Performance to Detect Upper Tract Disease

Of the 2,166 patient who underwent RBUS 14 (0.6%) had renal cell carcinoma and 7 (0.3%) had UTUC. CTU was performed in 1,692 patients, of whom 35 (2.1%) had renal cell carcinoma and 18 (1.1%) had UTUC. The table shows the diagnostic ability of RBUS and CTU to detect upper tract disease.

**Table:** Comparison of renal and bladder ultrasound, computerized tomography urogram and cystoscopy to diagnose bladder and renal cancer, and upper tract urothelial carcinoma

Reference Standard (diagnostic test)	No. Pts	Median % Diagnostic Accuracy (IQR)				AUC
		Sensitivity	Specificity	Pos Predictive Value	Neg Predictive Value	
Upper tract urothelial Ca histopathological confirmation:						
Renal + bladder ultrasound	2,166	14.3 (0.9-49.4)	100 (99.8-100.0)	50.0 (3.8-96.2)	99.7 (99.4-99.9)	0.571
Computerized tomography urogram	1,692	–	99.6 (99.2-99.8)	72.0 (52.8-86.9)	–	–
Renal Ca histopathological confirmation:						
Renal + bladder ultrasound	2,166	85.7 (62.1-97.5)	99.2 (98.8-99.5)	41.4 (24.8-59.5)	99.9 (99.7-100.0)	0.925
Computerized tomography urogram	1,692	–	99.9 (99.6-100.0)	94.6 (84.2-99.1)	–	–
Computerized tomography urogram to diagnose renal calculi (renal + bladder ultrasound)	475	34 (21.9-47.7)	97.9 (96.2-99.0)	65.4 (46.3-81.6)	92.7 (90.0-94.8)	0.659
Bladder Ca histopathological confirmation:						
Renal + bladder ultrasound	2,166	50.7 (42.7-58.7)	99.3 (98.9-99.6)	84.3 (75.8-90.8)	96.5 (95.6-97.2)	0.750
Unoptimized renal + bladder ultrasound excluded	2,090	63.6 (54.7-71.9)	99.3 (98.9-99.6)	84.3 (75.8-90.8)	97.9 (97.2-98.4)	0.814
Computerized tomography urogram	1,692	80.5 (74.8-85.4)	97.0 (96.1-97.8)	79.3 (73.6-84.4)	97.2 (96.3-98.0)	0.887
Unoptimized computerized tomography urogram excluded	1,615	83.6 (78.1-88.3)	97.0 (96.1-97.8)	80.0 (74.2-85.0)	97.7 (96.8-98.4)	0.903
Cystoscopy	3,556	–	98.3 (97.9-98.7)	84.0 (79.7-87.5)	–	–

RBUS identified 12 of 14 renal cancers (85.7%) and misclassified 1 renal cancer as UTUC, increasing the sensitivity of cancer detection to 92.9% with 99.9% NPV. The sensitivity of RBUS to detect UTUC was poor at 14.3%. Three cases were misclassified as renal cancer and 1 UTUC diagnosed on RBUS was shown to be renal cancer on histology, suggesting 62.5% sensitivity to detect cancer with a NPV of 99.9%.

Given that CTU suspicious for renal cancer or UTUC was a trigger for nephrectomy or renal biopsy, the sensitivity and NPV of CTU could not be determined. The PPV of CTU to diagnose renal cancer was 94.6% and 2 lesions were benign. CTU had 72.0% PPV to diagnose UTUC and 19 suspected UTUC cases were correctly identified. Three suspected UTUC cases were histologically confirmed as renal cancer, suggesting a cancer PPV of 88.0%. Ureteroscopy with or without biopsy did not confirm cancer in 3 cases. The diagnostic performance of RBUS to identify renal calculi was poor when CT was the reference standard with 34.0% sensitivity, 97.9% specificity, 65.4% PPV and 92.7% NPV.

### Renal and Bladder Ultrasound, Computerized Tomography Urogram and Cystoscopy Diagnostic Ability to Identify Bladder Cancer

The table shows the diagnostic ability of RBUS, CTU and cystoscopy for detecting bladder cancer. The diagnostic accuracy of RBUS to identify bladder cancer included 50.7% sensitivity, 99.3% specificity, 84.3% PPV and 96.5% NPV. CTU was better than RBUS at identifying bladder cancer. CTU had 80.5% sensitivity, 97.0% specificity, 79.3% PPV and 97.2% NPV to identify bladder cancer. When excluding suboptimal scans, the diagnostic ability of RBUS and CTU to detect bladder cancer improved.

The sensitivity and NPV of cystoscopy could not be determined because patients with normal flexible cystoscopy findings were discharged home without followup cystoscopy. Using histopathological confirmation of tumor as the reference the specificity of flexible cystoscopy was high at 98.3% with a PPV of 84.0%.

## Discussion

We report that the incidence of upper tract cancer in patients who presented with hematuria was low. Upper tract cancer was identified in 50 patients (2.2%) who presented with macroscopic hematuria, of whom 1.4% had renal cancer and 0.8% had UTUC, and in 5 patients (0.4%) who presented with microscopic hematuria, including renal cancer in 0.4% and UTUC in 0%. RBUS can identify suspicious renal cancer. One cancer was misclassified as UTUC for a sensitivity of 92.9%. However, RBUS only had 62.5% sensitivity to identify suspected UTUC, including 3 cancers diagnosed as renal cancer and 1 UTUC which was renal cancer on histology. RBUS missed 3 of 8 UTUC cases. The fact that no UTUC was identified after a presentation of microscopic hematuria suggests that RBUS should be done to assess the upper urinary tract in patients who present with microscopic hematuria.

The role of cystoscopy to diagnose bladder cancer remains the gold standard. Cystoscopy has 98.3% specificity and 83.9% PPV.^16^ Conventional imaging modalities cannot replace cystoscopy. Even after excluding suboptimum scans the accuracy of RBUS to detect bladder cancer was poor with 63.6% sensitivity and 99.3% specificity. CTU had a higher diagnostic accuracy to identify bladder cancer, including 83.6% sensitivity and 97.0% specificity, but it was not sufficient to replace cystoscopy.

It was estimated that the incidence of microscopic hematuria is as high as 2.5% of the population and it increases to as high as 18% in male patients 70 years old or older.^17^, ^18^ However, most of these cases do not have a sinister identifiable cause of microscopic hematuria. CTU was shown to be superior to RBUS for identifying UTUC.^1^, ^7^ RBUS may miss ureteral tumors too small to cause luminal occlusion. In turn this results in a false-negative finding because no hydronephrosis is identified, which would otherwise prompt further imaging. The operator dependent nature of RBUS may also miss small renal pelvis UTUCs. While CTU is superior for identifying UTUC, the risk of UTUC in patients who present with microscopic hematuria is rare, suggesting that there is no benefit for CTU over RBUS.^7^

RBUS was shown to detect renal cancer with high sensitivity, although 14 cases had false-positive results. In these false-positive cases a second scan would be done, typically renal protocol CT, which would better characterize the renal mass. Thus, the approach to cystoscopy with RBUS instead of CTU to investigate the upper tract of patients who present with microscopic hematuria should be the preferred upper tract imaging of choice.

We acknowledge that RBUS has poor sensitivity for identifying renal calculi. Therefore, we propose that patients who present with symptoms suggestive of renal colic such as flank pain would benefit from RBUS with noncontrast CT of the kidneys, ureters and bladder or CTU. We acknowledge that replacing CTU with RBUS in patients with microscopic hematuria would potentially miss asymptomatic renal calculi when no hydronephrosis presents with microscopic hematuria. We believe that such patients would be uncommon and identifying them would be at the expense of subjecting a high number to CTU which would yield negative results.

In an ideal world all patients should be investigated with the best diagnostic test available. However, the risk of adverse events, the low disease incidence in the specific patient cohort and the high cost of diagnostic testing suggest that this may not be warranted. Cases of microscopic hematuria have a 0% disease specific incidence of UTUC and are below the 3% threshold for diagnostic investigation used by the NICE (National Institute for Health and Care Excellence) and the 1% suggested by the AUA.^3^, ^4^ Additionally, while the risk of an adverse reaction to iodinated contrast medium is low, it can be life threatening.^11^ The ionizing radiation from CTU is 4 mSv, which is 200 times that of a standard chest x-ray.^19^ Also, cumulative exposure to ionizing radiation was shown to account for 0.6% to 0.9% of diagnosed cancers.^10^

Further, cost-effectiveness analysis recommended performing RBUS instead of CTU to evaluate patients with microscopic hematuria.^20^ A comparison of 4 diagnostic approaches, including CT alone, cystoscopy alone, CT with cystoscopy and RBUS with cystoscopy, suggested that the combination of RBUS with cystoscopy represents the most cost-effective combination at $53,810 per each cancer detected. Replacing RBUS with CTU would cost $6,480,484 per each cancer identified. It was estimated that using RBUS instead of CTU would result in a cost savings of $390 million, which is much needed in an era of escalating health care cost.^21^

The role of cystoscopy to visualize the bladder remains the gold standard. Even after excluding suboptimal scans a patient with a normal CTU or RBUS still requires cystoscopy due to the high risk of false-negative findings. This is similar to the diagnostic ability of FDA (Food and Drug Administration) approved urinary biomarkers to detect bladder cancer, which have a reported sensitivity of 57% to 82% and a specificity of 74% to 88%.^22^ While larger tumors would be easily identifiable, smaller tumors might be missed. It is likely that optimized CTU with the bladder well distended and contrast material which fully opacifies the bladder lumen would improve diagnostic accuracy. However, such scans may be difficult to achieve in clinical practice.

The majority of bladder lesions are considered cancer until proven otherwise. However, we report that a visual diagnosis of malignancy had 83.9% PPV following white light cystoscopy. In the setting of surveillance cystoscopy 99% of low grade bladder cancers were distinguishable from high grade cancers by urologists.^23^ Cystoscopy is operator dependent and when it is performed by a more experienced cystoscopist, specificity would be higher. Thus, it is essential that suspicious bladder lesions be biopsied due to the high likelihood of malignancy. Bladder biopsy could be performed by flexible cystoscopy at the initial diagnosis, which could reduce the need for general anesthesia.

There are several limitations to this study. While we did not identify any UTUC that presented as microscopic hematuria, it is plausible that these patients might have initially presented with microscopic hematuria if screening for microscopic hematuria had been performed, although this is not recommended by any consensus. While sonographers normally visualize the renal tract with the bladder distended to adequately visualize the bladder, this was not performed in all cases. Similarly bladder assessment was limited on some CTU scans when contrast medium did not opacify the bladder or the artefact was due to metalwork in the pelvis. To account for such suboptimal scans we excluded these scans to determine the diagnostic accuracy of imaging to identify bladder cancer. Additionally, we could not determine the sensitivity of cystoscopy since we were unable to determine whether tumors were missed because patients with normal cystoscopy findings were discharged home and did not undergo a repeat test.

## Conclusions

Our results suggest that CTU can safely be replaced by RBUS to image the upper tracts in conjunction with cystoscopy as part of investigations following a presentation of microscopic hematuria. The risk of UTUC in patients with microscopic hematuria is extremely low and RBUS can identify renal parenchymal cancer with high sensitivity. When renal calculi is suspected, noncontrast CT of the kidneys, ureters and bladder with RBUS or CTU is necessary. Cystoscopy remains the diagnostic test of choice to detect bladder cancer.

## Appendix DETECT I participating centers and investigators

W. S. Tan, P. Khetrapal, B. W. Lamb, A. Sridhar, F. Ocampo, H. McBain and J. D. Kelly, University College London Hospital; K. Baillie, K. Middleton and Principal Investigator D. Watson, James Cook University Hospital; H. Knight, S. Maher and Principal Investigator A. Rane, East Surrey Hospital; B. Pathmanathan, A. Harmathova and Principal Investigator G. Hellawell, London North West University Healthcare; S. Pelluri and Principal Investigator J. Pati, Homerton Hospital; A. Cossons, C. Scott and Principal Investigator S. Madaan, Darent Valley Hospital; S. Bradfield, N. Wakeford and Principal Investigator H. Mostafid, Royal Surrey County Hospital; A. Dann, J. Cook, M. Cornwell and Principal Investigator R. Mills, Norfolk and Norwich University Hospital; S. Thomas, S. Reyner, G. Vallejera, P. Adeniran and Principal Investigator S. Masood, Medway Maritime Hospital; N. Whotton, K. Dent, S. Pearson, J. Hatton, M. Newton, E. Heeney, K. Green, S. Evans and Principal Investigator M. Rogers, Northern Lincolnshire and Goole NHS Foundation Trust; K. Gupwell, S. Whiteley, A. Brown and Principal Investigator J. McGrath, Royal Devon and Exeter Hospital; N. Lunt, P. Hill and Principal Investigator A. Sinclair, Macclesfield Hospital; A. Paredes-Guerra, B. Holbrook and Principal Investigator E. Ong, North Devon District Hospital; H. Wardle, D. Wilson and Principal Investigator A. Bayles, University Hospital of North Tees; R. Fennelly, M. Tribbeck, K. Ames and Principal Investigator M. Davies, Salisbury District Hospital; J. A. Taylor, E. Edmunds and Principal Investigator J. Moore, East Sussex Healthcare NHS Trust; S. Mckinley, T. Nolan, A. Speed, A. Tunnicliff, G. Fossey, A. Williams, M. George, I. Hutchins, R. Einosas, A. Richards and Principal Investigator A. Henderson, Maidstone Hospital; B. Appleby, L. Kehoe, L. Gladwell, S. Drakeley, J. A. Davies and Principal Investigator R. Krishnan, Kent and Canterbury Hospital; H. Roberts, C. Main and Principal Investigator S. Jain, St James's University Hospital; J. Dumville, N. Wilkinson, J. Taylor and Principal Investigator F. Thomas, Doncaster Royal Infirmary; K. Goulden, C. Vinod and Principal Investigator E. Green, Salford Royal Hospital; C. Waymont, J. Rogers, A. Grant, V. Carter, H. Heap, C. Lomas and Principal Investigator P. Cooke, New Cross Hospital; L. Scarratt, T. Hodgkiss, D. Johnstone, J. Johnson, J. Allsop, J. Rothwell, K. Connolly and Principal Investigator J. Cherian, Pennine Acute Hospitals NHS Trust; S. Ridgway, M. Coulding, H. Savill, J. Mccormick, M. Clark and Principal Investigator G. Collins, Tameside General Hospital; K. Jewers, S. Keith, G. Bowen, J. Hargreaves, K. Riley and Principal Investigator S. Srirangam, East Lancashire Hospitals NHS Trust; A. Rees, S. Williams, S. Dukes and Principal Investigator A. Goffe, Dorset County Hospital; R. Mistry, J. Chadwick, S. Cocks, R. Hull, A. Loftus and Principal Investigator L. Dawson, Royal Bolton Hospital; Y. Baird, S. Moore, S. Greenslade, J. Margalef, I. Chadbourn, M. Harris and Principal Investigator J. Hicks, Western Sussex Hospitals NHS Foundation Trust; P. Clitheroe, S. Connolly, S. Hodgkinson, H. Haydock and Principal Investigator A. Sinclair, Stepping Hill Hospital; E. Storr, L. Cogley and Principal Investigator S. Natale, Derriford Hospital; W. Lovegrove, K. Slack, D. Nash and Principal Investigator K. Smith, King's Mill Hospital; J. Walsh, A. M. Guerdette, M. Hill and Principal Investigator D. Payne, Kettering General Hospital; B. Taylor, E. Sinclair, M. Perry and Principal Investigator M. Debbarma, Pinderfields Hospital; D. Hewitt and Principal Investigator R. Sriram, University Hospitals Coventry; A. Power, J. Cannon, L. Devereaux and Principal Investigator A. Thompson, Royal Albert Edward Infirmary; and K. Atkinson, L. Royle, J. Madine and Principal Investigator K. MacLean, Royal Cornwall Hospital.
