# Supplementary material for: Can Renal and Bladder Ultrasound Replace Computerized Tomography Urogram in Patients Investigated for Microscopic Hematuria?
Source: J Urol. 2018 Nov;200(5):973–80. doi: 10.1016/j.juro.2018.04.065 (PMC6179963; doi:10.1016/j.juro.2018.04.065)
Supplement: Supplementary Table [file mmc1.pdf]

Supplementary Table: Patient demographics according to type of hematuria

	All patients (n=3556)	Macroscopic hematuria (n=2311)	Microscopic hematuria (n=1245)	p value
Age (median, IQR)	67.7 (57, 76)	68.1 (56.4, 76.2)	67.0 (56.9, 75.0)	0.568
Gender, n (%):				<0.001
Male	2112 (59.4)	1607 (69.5)	505 (40.6)	
Female	1444 (40.6)	704 (30.5)	740 (59.4)	
Ethnicity, n (%):				0.235
Afro-Caribbean	51 (1.4)	36 (1.6)	15 (1.2)	
South Asian	86 (2.4)	57 (2.5)	29 (2.3)	
East Asian	15 (0.4)	8 (0.3)	7 (0.6)	
White	3080 (86.6)	2013 (87.1)	1067 (85.7)	
Mix	31 (0.9)	20 (0.9)	11 (0.9)	
Other	23 (0.6)	18 (0.8)	5 (0.4)	
Not known	271 (7.6)	159 (6.9)	111 (8.9)	
Smoking history, n (%):				0.739
Non-smoker	1528 (42.9)	991 (42.9)	537 (43.1)	
Current/ ex-smoker	1896 (53.2)	1240 (53.7)	656 (52.7)	
Not known	137 (3.8)	80 (3.4)	52 (4.2)	
Any urinary tract cancer, n (%)	354 (10.0)	315 (13.6)	39 (3.1)	<0.001
Bladder cancer, n (%)	288 (8.1)	255 (11.0)	33 (2.7)	<0.001
Renal cancer, n (%)	37 (1.0)	32 (1.4)	5 (0.4)	0.006
UTUC, n (%)	18 (0.5)	18 (0.8)	(0)	0.002
Renal calculi, n (%)	270 (7.6)	215 (9.3)	55 (4.4)	<0.001
